# Effects of a shared decision making intervention for older adults with multiple chronic conditions: the DICO study

**DOI:** 10.1186/s12911-023-02099-2

**Published:** 2023-03-01

**Authors:** Ruth E. Pel-Littel, Bianca M. Buurman, Marjolein H. van de Pol, Jos W. R. Twisk, Linda R. Tulner, Mirella M. Minkman, Wilma J. M. Scholte op Reimer, Julia C. M. van Weert

**Affiliations:** 1 Department of Internal Medicine, Section of Geriatric Medicine, Amsterdam UMC, Academic Medical Center, University of Amsterdam, Amsterdam, The Netherlands; 2 Vilans, Center of Expertise for Long-Term Care, Utrecht, The Netherlands; 3 ACHIEVE, Center of Applied Research, Faculty of Health, Amsterdam University of Applied Sciences, Amsterdam, The Netherlands; 4 Department of Primary and Community Care, Radboud University Medical Center, Nijmegen, The Netherlands; 5 Department of Epidemiology and Data Science, Amsterdam UMC, Vrije Universiteit Amsterdam, Amsterdam, The Netherlands; 6 Department of Geriatric Medicine, OLVG, Amsterdam, The Netherlands; 7 TIAS School for Business and Society, Tilburg University, Tilburg, The Netherlands; 8 Department of Cardiology, Amsterdam UMC, Academic Medical Center, University of Amsterdam, Amsterdam, The Netherlands; 9 Amsterdam School of Communication Research/ASCoR, University of Amsterdam, PO Box 15791, 1001 NG Amsterdam, The Netherlands

**Keywords:** Shared decision making, SDM, Preparatory tool, Communication training, Geriatricians, Older patients, Multiple chronic conditions, Informal caregivers, Pragmatic trial

## Abstract

**Background:**

To evaluate the effects of a shared decision making (SDM) intervention for older adults with multiple chronic conditions (MCCs).

**Methods:**

A pragmatic trial evaluated the effects of the SDM^MCC^ intervention, existing of SDM training for nine geriatricians in two hospitals and a preparatory tool for patients. A prospective pre-intervention post-intervention multi-center clinical study was conducted in which an usual care group of older patients with MCC and their informal caregivers was included before the implementation of the intervention and a new cohort of patients and informal caregivers after the implementation of the intervention. SDM was observed using the OPTION^MCC^ during video-recorded consultations. Patient- and caregivers reported outcomes regarding their role in SDM, involvement, perceived SDM and decisional conflict were measured. The differences between groups regarding the level of observed SDM (OPTION^MCC^) were analyzed with a mixed model analysis. Dichotomous patient-reported outcomes were analyzed with a logistic mixed model.

**Results:**

From two outpatient geriatric clinics 216 patients with MCCs participated. The mean age was 77.3 years, and 56.3% of patients were female. No significant difference was found in the overall level of SDM as measured with the OPTION^MCC^ or in patient-reported outcomes. However, at item level the items discussing ‘goals’, ‘options’, and ‘decision making’ significantly improved after the intervention. The items discussing ‘partnership’ and ‘evaluating the decision-making process’ showed a significant decrease. Fifty-two percent of the patients completed the preparatory tool, but the results were only discussed in 12% of the consultations.

**Conclusion:**

This study provides scope for improvement of SDM in geriatrics. Engaging older adults with MCCs and informal caregivers in the decision making process should be an essential part of SDM training for geriatricians, beyond the SDM steps of explaining options, benefits and harms. More attention should be paid to the integration of preparatory work in the consultation.

**Supplementary Information:**

The online version contains supplementary material available at 10.1186/s12911-023-02099-2.

## Background

In the care for older adults with multiple chronic conditions (MCCs), shared decision making (SDM) can be used to reach health decisions that are in line with the personal goals and preferences of the patient [1–5]. SDM is defined as “an approach where clinicians and patients share the best available evidence when faced with the task of making decisions, and where patients are supported to consider options to achieve informed preferences” [6]. SDM among older adults with MCCs has many benefits including a better understanding of harms and benefits, increased risk perception and less decisional conflict [7, 8]. However, the process of SDM is more complex in populations with older adults with MCCs than in younger populations for three main reasons.

First, the concept of MCCs is difficult to handle within the mainstream SDM models, which were developed for treatment decisions that aim to reach specific disease-specific outcomes for one disease. However, for many older adults with MCCs, personal health outcomes such as maintaining (functional) independence, reducing symptom burden, improving emotional health and the safety of treatment are often more important than single disease-specific outcomes [5]. To identify personal health outcomes, the recently published action steps for decision making for older adults with MCCs (based on the American Geriatrics Society (AGS) Guiding Principles for the Care of Older Adults with Multimorbidity) state that optimal care for older adults with MCCs should include eliciting and incorporating patient priorities into medical decision making [9, 10]. These action steps are well addressed within the ‘Dynamic model for SDM in frail older adults’, since this model emphasizes the clarification of personal goals, values and preferences, as well as the discussion of preferred roles and decision making capacities in decision making as an answer to the specific requirements needed for SDM with older adults with MCCs [11]. This model, validated by both health professionals and older adults with MCCs, states that adequate decisions are facilitated when they are based on the personal health outcome goals as prioritised by patients. To this aim, the model addresses specific issues for SDM in older adults such as a broad ‘holistic’ assessment of the patient’s problems, an exploration of important health outcome goals and discussing the decision-making capacity and preferences of the patient and the informal caregiver [11]. Specifically, the model introduces two preliminary steps, ‘Preparation’ and ‘Goal talk’, in addition to the generally known three steps of SDM models, i.e. ‘Choice talk’, ‘Option talk’ and ‘Decision talk’ [6]. Moreover, one additional last step, ‘Evaluation’, is added in the model. However, the feasibility and effectiveness of using this model in daily practice has not yet been studied. Second, for older adults with MCCs, it might be harder to participate in SDM due to the high prevalence of cognitive impairment, frailty, low health literacy and anxiety in this population [12–20]. This implies that health professionals must put extra effort to engage older adults with MCCs in SDM. Third, very few SDM models address the involvement of informal caregivers in the decision making process, while SDM with older adults with MCCs is often a triadic process, involving not only patients and health professionals but also informal caregivers.

The DICO study (Decision making In Complex Older populations) focused on solving these problems. In this study, we have identified a number of knowledge gaps on how SDM can facilitate healthcare conversations between health professionals, older adults with MCCs and their informal caregivers. Following the Medical Research Council (MRC) framework for developing complex interventions [21], we developed the SDM^MCC^ intervention to improve SDM for older adults with MCCs and their informal caregivers. In the development phase (phase 1) a theoretical basis for the SDM^MCC^ intervention was identified, through a systematic literature review of barriers to and facilitators of SDM in older patients with MCCs [22]. This was expanded with empirical research on how MCCs influence personal views on the ageing process [23] and how the TOPICS-MDS, a Patient Reported Outcome Measure (PROM) for healthcare conversations targeting older adults with MCCs, can be used as input for how older patients with MCCs can be empowered to partake in communication during consultations [24]. We also conducted a video observation study of usual care medical geriatric consultations [25]. After the development of the on these insights based SDM^MCC^ intervention, the feasibility of the intervention was tested (phase 2). Adaptations were done based on the results of the pilot-tests. Next, the intervention was implemented in two hospitals (phase 3). The current paper reports of the evaluation phase (phase 4). The objectives of the study were to evaluate the effects of the implemented SDM^MCC^ intervention in two hospitals on (1) observed SDM during consultations and (2) patient-reported outcomes.

We hypothesized that:

1. The implementation of the SDM^MCC^ intervention would result in improvement of post-intervention SDM during consultations as compared to SDM during pre-intervention consultations.
2. The implementation of the SDM^MCC^ intervention would result in increased patient-reported outcomes among older adults with MCCs and their informal caregivers after the implementation of the intervention as compared to patient-reported outcomes before the implementation of the SDM^MCC^ intervention.

In addition, a process evaluation was conducted to evaluate the implementation of the intervention.

## Methods

### Design, setting and locations

A pragmatic trial design was carried out at the geriatric outpatient departments of two Dutch hospitals in Amsterdam: (1) the department of Geriatric Medicine of the Amsterdam UMC, location Academic Medical Center (AMC) and (2) the outpatient clinic of Geriatric Medicine of the Medical Center Slotervaart (MC SLV). At the time of the study, two researchers (RPL and BB) were affiliated to the first department and a third one (LT) was affiliated to the second department. Pragmatic trials may test the same intervention as an explanatory trial, but they are conducted in real-world clinical practice settings. Specifically, we performed a prospective pre-intervention post-intervention multi-center clinical study to investigate the effect of the implementation of the SDM^MCC^ intervention. SDM during consultation and patient- and informal caregivers reported outcomes regarding perceived and preferred roles in SDM and in patient involvement, perceived SDM and decisional conflict were measured during a first period of 15 months before implementation of the SDM^MCC^ intervention (pre-intervention; April 2016–June 2017). The results were compared with results measured during a second period of 9 months after implementation (post-intervention; Oct 2017–June 2018). In the pre-intervention (usual care) group a video-observational study was conducted among ten geriatricians who were consulted by 108 geriatric patients. Next, the SDM^MCC^ intervention was implemented through a training for nine of these geriatricians (one dropped out) of the AMC (n = 4) and the MC SLV (n = 5) and by sending geriatric patients a preparatory tool to prepare for the consultation (see “Intervention” section). Subsequently, a second video-observational study was conducted among the same nine geriatricians consulted by a new group of geriatric patients (n = 108). For the reporting of this trial the extended CONSORT statement for pragmatic trials is followed [26, 27]. The local institutional review board waived the requirement to obtain approval for this study (W16_107#16.125, W17_284#17.336).

### Eligibility criteria for participants

To be eligible for the study, patients had to meet the following inclusion criteria: (1) being scheduled for a consultation with a geriatrician in one of the geriatric outpatient clinics of the two participating hospitals; (2) sufficient mastery of the Dutch language, and (3) a life expectancy of more than 3 months. Exclusion criteria were (1) having a severe stage of dementia (MMSE ≤ 15), according to the medical file, and (2) patient already included in study during previous visit. Hence, each patient could only participate once. Informal caregivers should be 18 years or older. There were no (other) inclusion or exclusion criteria for geriatricians or informal caregivers.

### Intervention

The SDM^MCC^ intervention was composed of an SDM^MCC^ training for geriatricians and a preparatory tool for the older adults and their caregivers (See Additional file 1). The rationale, goals, and a detailed description of the SDM^MCC^ intervention including the choices that were made about core components of the intervention have been presented in detail in a previous article in this journal [28]. In summary, the SDM^MCC^ intervention was based on our literature review of barriers and facilitators to SDM as experienced by health professionals, older adults with MCC and their informal caregivers [22] and our empirical research through a qualitative content analysis of structured interviews [23], a Delphi study [24] and a video observation study of (usual care) medical geriatric consultations [25]. Both the training for geriatricians and the preparatory tool were developed in a co-creation process with end-users (geriatricians, older patients with MCCs and informal caregivers) and tested in a feasibility study that consisted of several rounds [28]. After each round, adjustments were made based on the results of the feasibility tests. This resulted in the final SDM^MCC^ training and preparatory tool.

The SDM^MCC^ training for geriatricians was carried out between July and October 2017 and consisted of a 4-h intensive training session including theory and role playing with a professional training actor. The aim of the SDM^MCC^ training was to develop skills among geriatricians to involve older adults and their caregivers in SDM and to practice the six-step 'Dynamic model for SDM with frail older patients', as well as to learn how to explore personal goals related to quality of life and how to form a partnership with the patients and caregivers. In addition, the patient preparatory tool was presented and discussed during the training. Six months after the SDM^MCC^ training, an individual feedback session was offered in which the trainer and the geriatricians reflected together on SDM-skills in (video recorded) real consultations.

The preparatory tool for older patients with MCC and their informal caregivers was a leaflet consisting of four pages. Page 1 was an explicit invitation to partake in SDM and the acknowledgement that the patient’s own knowledge is valuable. Page 2 included an encouragement to share information about daily and social functioning and quality of life as well as an exploration of possible goals. Page 3 supported the older adult to prepare for the conversation with the geriatrician by means of an open question ‘what would you like to discuss with the doctor’ as well as by providing ‘example questions’ about exploring their options. These elements were based on literature about patient empowerment in SDM, such as underlying principles of Question Prompt Lists [29] and the ‘Ask 3 Questions’ campaign in the U.K. [30]. Page 4 focused on the informal caregiver by recognizing partnership and the potential burden of informal care and assessing informal caregiver burden. Furthermore, informal caregivers were also invited to share their concerns.

Details regarding the implementation of the training and the preparatory tool are in Additional file 2.

### Recruitment

Before the start of the study, the first author gave a presentation in a team meeting of the geriatricians in each of the two hospitals to explain the study and the teams agreed to participate. The first time that one of the geriatrician’s patients gave consent to participate in the study, the respective geriatrician gave also written informed consent. One week before each geriatric outpatient clinic in both hospitals, the schedule of the outpatient clinic was reviewed and potential eligible patients were called and informed about the study by a research assistant or the main researcher (RPL). If they were interested to participate in the study, they received an information package by mail, existing of a patient information letter with informed consent form. The post-intervention group also received the preparatory tool. One day before the consultation, the patients were called again and given the opportunity to ask questions. If they agreed to participate, they completed a pre-consultation questionnaire, which was part of the information package they had received, at home or just before consultation in the waiting room. Both the patient and the informal caregiver provided written informed consent. After this was obtained, the consultation was video recorded. A research assistant was present in the waiting room to assist patients and/or informal caregivers with the questionnaire, if necessary, and to start the video recording in the consultation room, but left the room during the consultation. Hence, the observers were not present during the actual consultation. The consultations were video recorded to enable rating by different observers. After the consultation, both the patient and the informal caregiver completed a post-consultation questionnaire. Data concerning comorbidities were retrieved from the patients’ medical records. The geriatricians completed a baseline questionnaire and a short post-consultation questionnaire. None of the participants received an honorarium for participation.

### Outcomes

#### Primary outcome

The primary outcome measure of this study was the level of observed SDM during clinical consultations, as measured with the OPTION^MCC^. The OPTION^MCC^ was an adapted version of the Observer OPTION-5 (from now on called OPTION-5) and recently developed to be able to measure triadic decision making in older adults with MCCs [25]. The adapted metric builds on the ‘Dynamic model for SDM in frail older adults’ [11] and contains 7 items that measure the competences of geriatricians and the level of participation among older adults and their caregivers. The OPTION-5 contains 5 items and includes most, but not all, competences described in the 'Dynamic model for SDM in frail older patients’ [31]. Therefore, the items ‘goal talk’ and ‘evaluation talk’ were added to the OPTION-5. Since our observations were limited to the consultation, the first step of the ‘Dynamic model for SDM in frail older adults’ (‘preparation’) that has to be taken before the consultation was not included in the OPTION^MCC^. The seven OPTION^MCC^ items are:

- Item 1: “Goal Talk” includes identifying the discussion partner, identifying the patient’s values and discussing the goals of care.
- Item 2: “Option Talk (1)” refers to explaining that there are more options.
- Item 3: “Team Talk” focuses on supporting deliberation and forming a partnership with the patient.
- Item 4: “Option Talk (2)” refers to informing the patient about eligible options.
- Item 5: “Decision Talk (1)” is about eliciting the patient's preferences.
- Item 6: “Decision Talk (2)” is about integrating the preferences and making the decision.
- Item 7: “Evaluation Talk” is about evaluating the SDM process with the patient and formulating a treatment plan.

The scores are allocated to increasing levels of achievement for the described competence of the geriatrician (range 0–4, transformed 0–100). The level of patient and informal caregiver participation is rated on three levels: (0) no participation, (1) responsive participation and (2) active participation.

#### Secondary outcomes

The following patient-reported outcomes were measured as secondary outcomes:

1. *Match between preferred and perceived role in decision making* of patients and informal caregivers was measured before (preferred) and after (perceived) the consultation using an adapted version of the Control Preference Scale. The adapted scale contained seven response statements, which were divided among three categories: (1) an active role (patient- and/or informal caregiver-controlled), (2) a passive role (practitioner-controlled), and (3) a shared role (collaborative) (see Additional file 3), with the informal caregiver as a partner in decision making [32, 33]*.* The match between the participants' preferred and perceived role was used as patient-reported outcome. Participants who had identical scores on the preferred and perceived role were categorized as having matched preferences.
2. *Match between preferred and perceived involvement* of patients and caregivers in their care was measured with Patients’ Perceived Involvement in Care Scale (PICS) before (preferred) and after (perceived) the consultation [34, 35]. Participants were asked to indicate the importance of eight statements concerning the upcoming consultation. The statements were measured on a 4-point Likert scale, with options ranging from ‘*Not important*’ (1) to ‘*Very important*’ (4). These preference scores were divided into high (scores 3 and 4 = 1) and low (scores 1 and 2 = 0) importance for involvement for each item. Furthermore, a ‘*Not applicable*’ option was included for statements that were not relevant to the consultation. To measure perceived involvement, participants could indicate whether they (0) did not perform or (1) did perform the behavior during their consultation. The total score ranged from 0 to 8 for both scale [35]. The match between the participants' preferred and perceived involvement was used as patient-reported outcome and calculated by subtracting the perceived participation score from the preferred participation score. Participants with discrepancy scores between − 2 and 2 were categorized as having matched preferences. Participants with discrepancies <  − 2 or > 2 were categorized as having unmatched preferences.
3. *The level of perceived SDM* was measured with CollaboRATE. Responses to each item range from 0 (no effort was made) to 9 (every effort was made). CollaboRATE scores are calculated as the proportion of participants who report a score of nine on each of the three CollaboRATE questions [36–38].
4. *Decisional conflict* was measured with the Decisional Conflict Scale (DCS), consisting of 16 items clustered in 5 subscales: ‘informed’, ‘values clarity’, ‘support’, ‘uncertainty’ and ‘effective decision’ [39–41]. All items are measured on a 5-point Likert scale [39]. The total score varies between 0 (no decisional conflict) and 100 (extremely high decisional conflict) [39].

#### Background characteristics

Patients’ and caregivers’ baseline characteristics included: age, gender, education (low, middle, high), living situation and health literacy [42]. Clinical characteristics included frailty [43], polypharmacy and comorbidity [44].

#### Process evaluation

For each consultation, geriatricians reported the most important problem presented by the patient and the decision. Geriatricians also indicated whether there were more options available and, if so, whether these options were equal; meaning subject to preference-sensitive decisions [45] (see Table 1). In the post-intervention questionnaire, patients and caregivers were asked whether they received the preparatory tool before the consultation and whether they had completed it. In the video observations it was observed whether the tool was used or referred to.

**Table 1:** Baseline characteristics by group

Characteristics	All patients (N = 213)	Pre-intervention	Post-intervention	Pre-intervention vs post-intervention patients
		Usual care patients (n^a^ = 105)	Intervention patients (n^a^ = 108)	*p* value
Mean age in years (SD)	77.3 (7.9)	78.0 (8.2)	76.5 (7.4)	.56
Female sex (n, %)	120 (56.3)	55 (52.4)	65 (6.2)	.25
Level of education				.45
Low (n, %)	31 (15.2)	19 (18.8)	12 (11.1)	
Middle (n, %)	118 (57.8)	58 (57.5)	60 (55.6)	
High (n, %)	55 (27.0)	23 (22.8)	32 (29.6)	
Living situation				.63
Independent, alone (n, %)	91 (43.5)	41 (4.6)	50 (46.3)	
Independent, with others (n,%)	113 (54.1)	58 (57.5)	55 (5.9)	
Home for the elderly (n, %)	5 (2.4)	2 (2.0)	3 (2.8)	
Health literacy (SAHL-D22)^b^ (mean, SD)	11.8 (6.9)	1.7 (6.9)	12.9 (6.8)	.55
Polypharmacy^c^ (≥ 4) (n, %)	137 (64.3)	65 (61.9)	72 (66.7)	.93
Frailty (GFI)^d^ mean, SD	4.3 (2.5)	4.4 (2.6)	4.2 (2.4)	.69
Comorbidity (CCI)^e^ mean, SD	1.9 (1.9)	2.09 (1.8)	1.65 (1.9)	.09
Duration consultations (in min) mean (SD)	38.7 (33.5)	40.9 (26.8)	36.6 (38.6)	.37
Main problem (according to geriatrician) (n, %)				.29
Cognition/dementia	98 (45.2)	52 (48.0)	46 (41.4)	
Osteoporosis	27 (12.4)	9 (8.0)	18 (16.2)	
Falls/mobility	17 (7.8)	10 (9.0)	7 (6.3)	
Depression	9 (4.1)	3 (3.0)	6 (5.4)	
Other (< 5%)	66 (3.4)	34 (32.0)	32 (3.7)	
Most frequently discussed decisions^f^ (according to geriatrician) (n,%)				.32
Additional diagnostics	69 (22.6)	31 (24.2)	38 (21.5)	
Follow-up	66 (21.6)	27 (21.1)	39 (22.0)	
Medication	57 (18.7)	28 (21.8)	29 (16.4)	
Referral to primary care	49 (16.1)	19 (14.8)	30 (16.9)	
Lifestyle	29 (9.5)	12 (9.4)	17 (9.6)	
Consultation other hospital specialist	26 (8.5)	8 (6.2)	18 (1.2)	
Other	9 (3.0)	3 (2.3)	6 (3.4)	
More options were available (according to geriatrician) (n, %)	167 (78.4)	82 (87.2)	85 (78.7)	.17
If so, options were considered equal (according to geriatrician) (n, %)	83 (39.0)	36 (43.3)	47 (53.4)	.42

^a^Three patients were excluded after analysis, due to too much missing data (see flow chart) and n varies slightly due to missing data

^b^Health literacy: SAHL-D22 (score 0–22; a higher score indicates higher health literacy)

^c^Polypharmacy: use of ≥ 4 different medications

^d^Frailty: GFI (score 0–15; score > 4 indicates frailty)

^e^Comorbidity: CCI. A higher CCI-score (CCI-score > 5) is associated with higher morbidity and mortality

^f^More decisions in 1 consultation were possible

* < .05; ** < .01; *** < .001; *SD* standard deviation

### Sample size

There was no prior data on our primary outcome ‘the level of observed SDM during clinical consultations’, as measured with the OPTION^MCC^. Therefore, we based our sample size on Barr et al. (2015), who expected that with 90% power and an alpha level of .05, a sample size of 100 participants per arm would be needed to detect a minimum of a 3.5 point difference in OPTION-5 scores [46].

### Allocation, blinding and inter-rater agreement

Since we compared a pre-intervention group with a post-intervention group of patients and informal caregivers, there was no other randomization procedure than the timing of inclusion. Participants were included by the main researcher or by research assistants. These researchers and participating geriatricians were not blind for the intervention. Patients and informal caregivers in both the pre-intervention and the post-intervention group were informed that the aim of the study was to better understand decision-making in the outpatient geriatrics clinic. Hence, they were blind. The videos were assessed by three trained observers [25]. To avoid bias in rating the videos before and after the intervention, we involved a fourth, independent observer who assessed 20 videos. Inter-rater agreement was assessed using the intraclass correlation coefficient (ICC), calculated with a two-way mixed absolute agreement model. ICC scores were .77, .77, and .88 (geriatricians, patients, and caregivers, respectively), which indicated substantial levels of agreement.

### Statistical analysis

We used descriptive statistics to summarize personal, clinical, and other outcomes of patients and caregivers. Descriptive statistics were also used to summarize consultation characteristics and the results of the process evaluation. The differences between groups regarding the level of observed SDM (OPTION^MCC^) were analyzed with a mixed model analysis. The mixed model included only the group variable and a random intercept for the geriatrician to adjust for the dependent observations within the geriatricians. The same was done for the patient-reported outcomes ‘level of perceived SDM’ and ‘decisional conflict’. For dichotomous patient-reported outcomes (i.e., match between preferred and perceived role in decision making resp. match between preferred and perceived involvement in care) a logistic mixed model analysis was used.

## Results

### Response

**Fig. 1:**
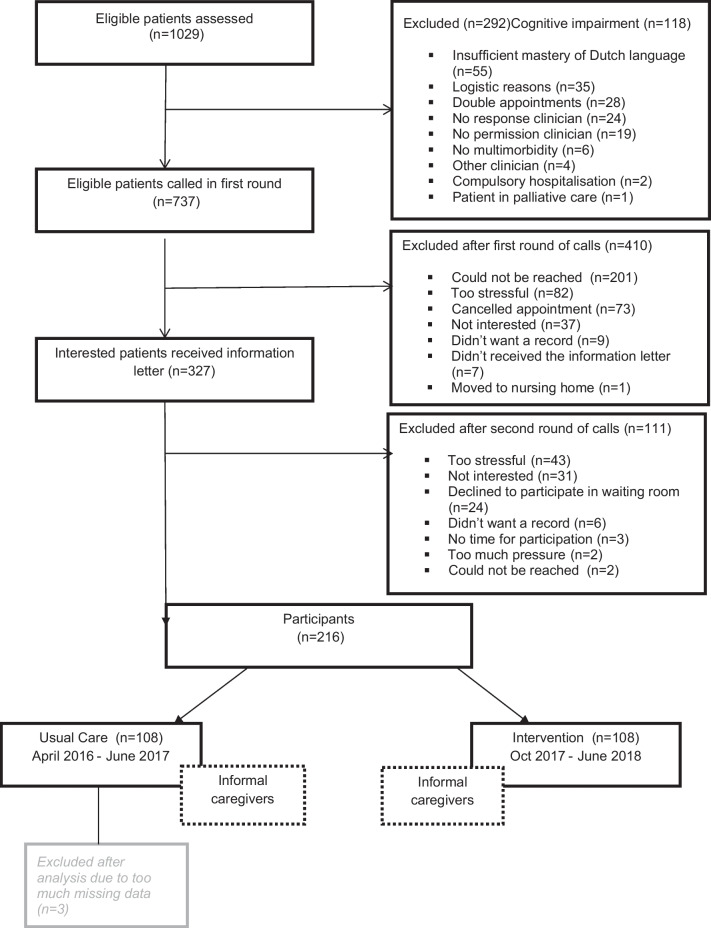
Flowchart of patient inclusion

Figure 1 presents the flow chart of the study. In the study period, 1029 older adults visited one of the two hospitals for a geriatric consultation with a geriatrician of which 216 geriatric patients with MCCs and their 133 informal caregivers participated in the study. The main reasons for exclusion or refusal to participate were that the patient could not be reached (n = 201), the patient found participation too stressful (n = 125), the patient had cognitive impairment (n = 118), the appointment was cancelled (n = 73), the patient was not interested (n = 68), or the patient had insufficient mastery of the Dutch language (n = 55). There were no significant differences regarding age and gender between the participating patients and non-responding patients. Of the 216 participating patients, 213 were included in the final analysis (108 in the pre-intervention group and 105 in the post-intervention group), due to too much missing data of three participating patients. We included all 133 informal caregivers in the final analysis, 68 in the pre-intervention group and 65 in the post-intervention group.

### Baseline characteristics

Table 1 summarizes the sociodemographic and clinical characteristics of patients and the characteristics of the consultations. The mean (standard deviation (SD)) age was 77.3 (7.9) years, and 56.3% were female. The main problem was cognition (45.2%), and the main decisions were about additional diagnostics (22.6%), follow-up (21.6%) and medication (18.7%). The mean duration of the consultations was 38.7 min (SD 33.5). The background characteristics of the caregivers are presented in Additional file 4.

### Level of observed SDM (primary outcome)

Table 2 shows the OPTION^MCC^ item response. Overall, there were no significant differences between the intervention group and the usual care group for the total mean OPTION^MCC^ scores. However, at the item level, we observed significant differences in 6 out of 7 subitem responses. For both geriatricians and patients, we found a significant improvement in item 1: goal talk (geriatricians *B* .32, 95% confidence interval (CI) .06; .58, patients *B* .27, 95% CI .10; .44) and item 4: option talk (geriatricians *B* .25, 95% CI .01; .48, patients *B* .22, 95% CI .04; .39). We also observed a significant improvement of patients in items 5 and 6 regarding decision talk (elicit preferences and decide together) (patients item 5 *B* .39, 95% CI .21; .57, item 6 *B* .24, 95% CI .07; .41). However, a significant decrease was observed for item 3: team talk (geriatricians *B* − .71, 95% CI − 1.01; − .40, patients *B* − .52, 95% CI − .72; − .33, caregivers *B* − .52, 95% CI − .72; − .33) and item 7: evaluation talk (geriatricians *B* − .46, 95% CI − .70; − .21, caregivers *B* − .32, 95% CI − .55; − .09).

**Table 2:** Observer OPTION^MCC^ outcomes for geriatricians, patients and informal caregivers

Geriatricians	Pre-intervention (n = 100^a^)	Post-intervention (105^a^)	*p* value	*B* (95%CI)
OPTION scores on subitems (mean, SD) range (0–4)				
Goal talk	1.6 (1.1)	1.9 (.9)	.02*	.32 (.06; .58)
Option talk: present options	1.8 (.9)	1.9 (.6)	.30	.11 (− .10; .33)
Team talk: form partnership	1.2 (1.2)	.5 (1.0)	< .001^***^	− .71 (− 1.01; − .40)
Option talk: discuss pro’s and con’s	1.6 (.9)	1.9 (.8)	.04^*^	.25 (.01;.48)
Decision talk: elicit preferences	1.8 (1.3)	2.0 (.9)	.22	.18 (− .11;.48)
Decision talk: decide together	1.6 (1.2)	1.8 (.9)	.16	.20 (− .08;.49)
Evaluation talk	1.4 (1.0)	.9 (.8)	< .001^***^	− .46 (− .70; − .21)
Total OPTION score	1.6 (.9)	1.6 (.5)	.88	− .01 (− .21;.18)
Total transformed OPTION score (0–100 score)	39.7 (21.4)	39.3 (13.7)	.88	− .37 (− 5.2; 4.45)

Patients	Usual care (n = 100^a^)	Intervention (105^a^)	*p* value	*B* (95%CI)
OPTION scores on subitems (mean, SD) range (0–2)				
Goal talk	1.2 (.7)	1.5 (.6)	.002**	.27 (.10; .44)
Option talk: present options	1.1 (.6)	1.3 (.6)	.10	.14 (− .03; .30)
Team talk: form partnership	.9 (.8)	.3 (.6)	< .001^***^	− .52 (− .72; − .33)
Option talk: discuss pro’s and con’s	1.1 (.7)	1.3 (.6)	.02*	.22 (.04; .39)
Decision talk: elicit preferences	1.2 (.7)	1.5 (.6)	< .001***	.39 (.21; .57)
Decision talk: decide together	1.1 (.7)	1.3 (.5)	.01**	.24 (.07; .41)
Evaluation talk	.9 (.7)	.8 (.6)	.09	− .15 (− .33; .02)
Total OPTION score	1.1 (.5)	1.1 (.4)	.19	.08 (− .04; .21)

Informal caregivers	Usual care (n = 68^a^)	Intervention (65^a^)	*p* value	*B* (95% CI)
OPTION scores on subitems (mean, SD) range (0–2)				
Goal talk	1.2 (.6)	1.3 (.7)	.45	.08 (− .04;.21)
Option talk: present options	1.2 (.7)	1.1 (.6)	.32	− .12 (− .34;.11)
Team talk: form partnership	1.0 (.8)	.2 (.5)	< .001^***^	− .78 (− 1.01; − .55)
Option talk: discuss pro’s and con’s	1.1 (.7)	1.2 (.7)	.57	.07 (− .16;.30)
Decision talk: elicit preferences	1.2 (.7)	1.3 (.7)	.12	.18 (− .05;.41)
Decision talk: decide together	1.1 (.7)	1.3 (.6)	.08	.20 (− .02;.42)
Evaluation talk	.9 (.7)	.5 (.7)	.01^**^	− .32 (− .55; − .09)
Total OPTION score	1.1 (.5)	1.0 (.5)	.20	− .10 (− .26;.05)

^a^n varies slightly due to missing data

* < .05; ** < .01; *** < .001; *SD *Standard deviation

### Patient-reported outcomes (secondary outcomes)

The results of the patient-reported outcomes are presented in Table 3. The match between preferred and perceived role in decision making was not significantly different between usual care patients and intervention patients nor for their informal caregivers. Additionally, we found no significant differences in the match between preferred and perceived participation in SDM and the level of perceived SDM in either group. Decisional conflict was low in the usual care and intervention groups, and no significant differences were found between these groups.

**Table 3:** Participant reported outcomes

	Patients pre-intervention	Patients post-intervention	*p* value	Caregivers pre-intervention	Caregivers post-intervention	*p* value
	Usual care patients (N^a^ = 105)	Intervention patients (N^a^ = 108)		Usual care informal caregivers (N^a^ = 68)	Intervention informal caregivers (N^a^ = 65)	
Preferred role in the decision making process (CPS) n (%)^b^			.63			.41
Active role	48 (48.5)	56 (51.9)		4 (6)	(1.6)	
Collaborative role	41 (41.4)	45 (41.7)		47 (7.1)	42 (67.7)	
Passive role	10 (1.1)	7 (6.5)		16 (23.8)	19 (3.6)	
Perceived role in the decision making process (CPS) n (%)^b^			.91			1.0
Active role	41 (52.5)	47 (53.4)		3 (5.4)	2 (4)	
Collaborative role	26 (33.3)	27 (3.7)		30 (54.5)	27 (54)	
Passive role	11 (14.1)	14 (15.9)		22 (40)	21 (42)	
Match preferred and perceived role in the decision making process (Match CPS) n (%)^c^	45 (57.7)	43 (48.9)	.26	34 (50)	35 (54)	.23
Preferred involvement in care; PICS) mean (SD)^d^	6.6 (1.8)	6.3 (1.9)	.30	6.9 (1.5)	6.6 (2.1)	.07
Perceived involvement in care (PICS) mean (SD)^d^	5.2 (2.8)	5.6 (3.6)	.66	5.3 (2.5)	5.6 (3.6)	.15
Match preferred and perceived involvement in care (Match PICS) n (%)^e^	44 (59.5)	46 (57.5)	81	29 (59.2)	23 (57.5)	.87
Level of perceived SDM (CollaboRATE) n (%)^f^	44 (56.4)	37 (43.0)	.09	23 (41.1)	19 (38.0)	.75
Decisional conflict (DCS) mean (SD)^g^	23.3 (2.9)	24.1 (19.7)	.85	22.9 (2.7)	21.5 (17.1)	.40

*SD* Standard deviation

^a^n varies due to missing data

^b^Decision roles: Adapted Control Preference Scale (CPS) with seven response statements, which are divided among three categories: (1) an active role (patient- and/or informal caregiver-controlled), (2) a passive role (practitioner-controlled), and (3) a shared role (collaborative)

^c^Match between preferred and perceived role in the decision making process: Match CPS: participants who had identical scores on the preferred and perceived roles were categorized as having matched preferences

^d^PICS: Patients’ Perceived Involvement in Care Scale (score range 0–8) higher scores indicating a higher preferred resp. perceived participation during the decision making process

^e^Match between preferred and perceived involvement in care: PICS Match: participants with discrepancy scores between − 2 and 2 were categorized as having matched preferences

^f^SDM: CollaboRATE % patients that have a top score, a higher % indicates a higher level of SDM

^g^Decisional Conflict: DCS: (score 0–100) a higher score indicates a higher level of decisional conflict

### Process evaluation

Additional file 5 presents the evaluation of the preparatory tool usage. Seventy-four (74/108; 68.5%) older adults remembered that they had received the preparatory tool. The tool was filled in by 56 older adults (56/108; 51.9%) of whom 26 (26/108; 24.1%) reported that they discussed the tool with an informal caregiver before the consultation. There were no significant differences in the total mean OPTION^MCC^ scores between the patient intervention group that had completed and used the preparatory tool and those that had not used the preparatory tool (see Additional file 6).

Table 4 shows the mean OPTION^MCC^ score of each geriatrician (range − 17.3 to 24.08). Of the nine geriatricians who participated both in the pre-intervention and in the post-intervention measurements, one received a higher mean overall OPTION^MCC^ score after the intervention (*p* =  < .01), one received a lower mean OPTION^MCC^ score (*p* = .01), and seven geriatricians showed no significant difference in their mean OPTION^MCC^ score after the intervention (range − 7.92 to 8.29). The one geriatrician with a lower score had a strongly deviating score (− 17.3) compared to the other eight geriatricians. When we considered this as an outlier, a subgroup analysis of the remaining eight geriatricians revealed a significant positive effect on the overall OPTION^MCC^ mean scores after the intervention (See Additional file 7).

**Table 4:** Total OPTION^MCC^ scores individual geriatricians (pre-intervention vs. post-intervention)

Geriatrician	Pre-intervention	Post-intervention	Change	*p* value
	Usual care (n = 10) (mean score)	(n = 9) (mean score)		
1	51.13	33.83	− 17.3	<.01**
2	40.36	38.49	− 1.87	.80
3	17.35	41.43	+ 24.08	< .01**
4	41.67	43.11	− .24	.84
5^a^	38.57			
6	53.87	45.95	− 7.92	.21
7	34.18	39.29	+ 5.11	.79
8	30.10	38.39	+ 8.29	.31
9	32.65	38.57	+ 5.92	.37
10	32.14	34.69	+ 2.55	.68

^a^Geriatrician 5 did not participate in intervention

* < .05; ** < .01; *** < .001

On an item level, this subgroup analysis showed a significant improvement for both geriatricians and patients on 5 of the 7 subitems: item 1: goal talk, item 2: option talk (present options), item 4: option talk, item 5 decision talk (preferences) and item 6 decision talk (decision). For items 5 and 6, we also found a significant improvement in the informal caregiver scores. Similar to the overall analysis, a negative significant effect was found for item 3: team talk (geriatricians, patients and caregivers) and item 7: evaluation talk (geriatricians and caregivers).

Finally, we noted that often there was more than one problem that was discussed during a consultation. Although we asked geriatricians to define the most important problem, the multitude of problems discussed sometimes complicated the ratings of the observers.

## Discussion

This study aimed to evaluate the effects of the SDM^MCC^ intervention for older adults with MCCs and their caregivers on observed SDM. The intervention consisted of SDM^MCC^ training for geriatricians and a preparatory tool for the older adults and their caregivers. We measured the level of SDM during clinical consultations with the OPTION^MCC^, and observed for three out of seven items a significant improvement after the intervention. These are the items that were about ‘discussing goals with patients’, ‘explaining the options’ and ‘making the decision’. However, on two items we observed a significant decline. These are the items about ‘discussing that the input of the patient is just as important as the input of the geriatrician (so-called partnership)’, and about ‘evaluating the decision-making process’. On average, the combination of improvement on some items and deterioration on others did not lead to improvement, i.e. the total score on the OPTION^MCC^ did not show a significant difference after the implementation of the SDM^MCC^ intervention. There are several possible explanations as to why we only found differences on item-level and not an overall difference between the pre-intervention and post-intervention measurements with the OPTION^MCC^. One explanation can be that geriatricians are forced to prioritize within the limited time of a consultation and that time taken for discussing goals, options and the actual decision making (the elements that increased) occurs at the expense of the other steps. Another explanation might be that discussing goals, options and the actual decision making are the easiest parts of SDM to improve rather than establishing a genuine partnership with the older adults in which everyone’s input is equally important and taking time to evaluate the decision-making process. This is in line with the findings of Driever et al. (2019) about physicians' preferred and perceived roles in SDM, reporting that hospital physicians focused more on discussing treatment options and gave less attention to actually involving the patient in the decision-making process [47]. Furthermore, although we did address all steps of the SDM^MCC^ model in the SDM^MCC^ training, the part where geriatricians practiced SDM may have been more focused on discussing goals, one of the items that were added to existing SDM models in the ‘Dynamic model for SDM in frail older adults’, than the topics engaging the patient in the decision making process and evaluation. In addition, evaluating the decision-making process can be tense because of the vulnerability of the geriatrician: ‘Did I discuss it with you properly as a doctor?’. Finally, we concur with Pieterse et al. (2019) that we might have to rethink the underlying relationship between the items and the construct that we measure; in other words—how are the SDM items related to each other [48]? And should all items be given the same weight or, for example, should ‘discussing goals’ be given more weight than ‘evaluation of the SDM process’? For the future, we think that it might be of added value to make the item goal talk, in our opinion one of the most important strengths of the ‘Dynamic model of SDM with frail older patients’, more explicit. Following their previous research on goals setting for older adults with MCCs in SDM, Vermunt et al. (2017; 2018) [4, 5] recently proposed an integrated, goal-based SDM model using a Goal Board to prioritize collaborative goals and align goals with interventional options. This model describes three goal levels: fundamental, functional and symptomatic. Fundamental goals are about what people hope for in life, or are afraid of. Functional goals address the activities one wants to be able to do or to carry on doing. Symptom or disease specific goals concern the symptoms of disease someone wants to change, for example less pain. For future research it is interesting to explore how this Goal Board could be used. For example, we are currently exploring if this goal-based SDM model could be integrated with the ‘Dynamic model of SDM for frail older patients’. It might also be worthwhile to consider incorporating time-weighting strategies in relation to the steps of the SDM while taking into account consultation time and the different topics the geriatrician discusses during consultations. To warrant patient engagement, it may be useful to incorporate elements of a conceptual framework for patient engagement in SDM models [49] in future research.

Regarding the total OPTION^MCC^ score, it is notable that the overall mean score of the OPTION^MCC^ (range 0–100) in the current study was higher both before and after the intervention than the mean scores of observer OPTION measurements in previous studies, as described in a review of 33 studies (mainly among general practitioners) that used the OPTION-12 [50]. According to the review, better implementation of the intervention and longer consultation durations were associated with higher scores on the OPTION-12 scale [50]. Since the mean duration of consultations was much longer in our study (38.7 min) compared to most studies in the review (median 13 min), this might partially explain our higher overall scores. Moreover, there were striking differences in OPTION^MCC^ scores between the nine participating geriatricians, even though they all followed the same SDM^MCC^ training. A sub-analysis showed that this variety was not associated with the use of the patient preparatory tool, the availability of more options, the equality of the options, or with the hospital setting. Because in particular the results of one geriatrician were very different from the other geriatricians, we performed a subgroup analysis without this geriatrician. This analysis showed that the overall results (total OPTION^MCC^ score) of the other doctors had improved significantly. This indicates that a good implementation is important, and seems to confirm the relationship between better implementation of the intervention and higher scores on the OPTION scale [50]. Only a few interventions for SDM target both healthcare professionals and patients [51]. Because training programs targeting both groups seem to benefit SDM more than interventions targeting only one of these groups [52, 53], our intervention focusing on both groups was justified. In our study, the 4-h training for geriatricians included one session of feedback per geriatrician of a recorded consultation (see Additional file 2); but repeated video-based individual feedback sessions could have strengthened the effects of the SDM^MCC^ training, as shown by Geiger et al. (2017) [54]. Similar conclusions were drawn by Geessink et al. (2017), who also trained clinicians according to a dynamic model of SDM with frail older persons [55].

The process evaluation of the preparatory tool (leaflet) showed that although patients and caregivers were mostly positive about the preparatory tool and the preparatory tool was often filled in by older patients and/or their caregivers, the tool was discussed in only 12% of the consultations, almost always at the initiative of the geriatrician. Hence, people that had put effort in completing the preparatory tool (e.g. selected questions from the examples in the leaflet) often experienced that their input was not incorporated in the conversation with the geriatrician. Research shows that implementing question prompt lists can have a counterproductive effect when the preparatory work is not acknowledged by the physician, because the patient’s expectations, i.e. discussing what (s)he has filled in, are not met [56]. Other recent reports emphasize that question prompt sheets, such as the one in our preparatory tool, have more effect when combined with training of the health professionals [29, 56]. Although we did include the preparatory tool in the training for geriatricians, we might have focused more on instructing the geriatrician on how to discuss the preparatory tool with the patients. In the follow-up projects, we therefore devote more attention to the role of the geriatrician in discussing the preparation that was done by patients and/or their caregivers by using the leaflet.

The preparatory tool for this study was developed in collaboration with end users, i.e., older adults with MCCs, thus addressing the specific needs of a population in which cognitive decline and low health literacy are very common. This might explain other aspects of the process evaluation. First, we believe that due to cognitive decline (the most common problem in the patient group), almost one-third of the patients did not remember receiving the preparatory tool. Second, of the patients who remembered receiving the tool, a large majority (56/74; 75.6%) was able to complete the tool, suggesting sufficient feasibility of the preparatory tool. Furthermore, almost two-thirds of the patient users were positive about the tool. However, no significant differences were identified for patients and informal caregivers regarding patient-reported outcomes. This indicates that there might be more efforts needed at the patients’ and informal caregivers’ side, both by further improvement of the preparatory tool, and by training patients and their caregivers better in how to use the tool and to prepare for consultations with healthcare professionals (see practical implications).

### Limitations

This pragmatic trial with video recordings of real-life consultations provided a unique insight into SDM with older adults with MCCs and their caregivers. However, there are limitations. Although we found no overall differences between the usual care and intervention groups, there might be some bias caused by an increasing awareness of SDM in Dutch hospitals and in society over time, raising the expectations of the older adults and their caregivers to be involved in SDM. Moreover, a significant number of eligible participants could not be included in the study, which means that we have to interpret our findings with caution. In reasons for not participating in the study, in total 125 participants found that participation would be too stressful and two participants found that they were under too much pressure. Although we took several measures to make participants feel comfortable, both by taking the time during the two telephone calls preceding the consultation and by taking the time in the waiting room just before and after the consultation, we could not avoid that participating in the study was more than these patients could handle. This might be explained because the study focused on vulnerable people for whom the hospital visit in itself could already be very tiring and stressful. This is a serious and difficult to solve limitation, in particular in studies like this one, in which participants are frail and have no established relationship yet with the healthcare professional, here a geriatrician, like they usually do have with for instance their GP. In addition, we experienced that scoring behavioral competences of SDM in geriatric consultations is difficult. The OPTION^MCC^ is designed to measure explicit SDM behavior in a quantitative way; nonetheless, we also observed immeasurable, implicit SDM behavior—for example non-verbal behavior of a geriatrician or an empathic, attentive way of listening, thus empowering older adults to express themselves. It would be interesting in further research to consider this aspect when using or developing SDM measurement instruments or to do additional qualitative analysis. Last, the real gain may be that the decisions made contribute better to the personal health goals of the patient. However, this gain is only visible in the longer term and we have not been able to measure such longer term results in the current study.

### Practical implications

As explained above, the SDM^MCC^ needs further development regarding several aspects of the SDM^MCC^ training (‘team talk’ and ‘evaluation’) and regarding the implementation of the patient preparatory tool in the consultations. To facilitate a larger group of health professionals in SDM, in particular geriatricians, creating interactive online scenarios in which consultations with older adults are translated into conversations with virtual trainings actors could be useful. In addition to the original training, an online SDM^MCC^ training has been piloted at the department of geriatrics in five other Dutch hospitals then the hospitals that participated in the study, and is now free available for all healthcare professionals (https://samenbeslissen.dialoguetrainer.com/). The online training includes three follow ups and contains a self-assessment for geriatricians, in which they are encouraged to assess their own audiotapes of real life consultations with an adapted practice version of the OPTION^MCC^. Furthermore, together with the Dutch Geriatric Society (NVKG) and the Dutch Nurses Society (V&VN Geriatrics & Gerontology) and the largest Dutch senior organisation KBO-PCOB, we initiated an implementation programme to facilitate both health professionals as well as older adults and their informal caregivers in SDM. This programme includes the TOPICS-MDS [24], or preferably the short version (TOPICS-SF), a questionnaire to be completed by older adults with MCCs, which gives an overview of the current status of an older person regarding personal health outcomes that most older adults consider important. The TOPICS-SF provides input for the ‘goal talk’, step 2 of the ‘Dynamic model of SDM in frail older patients’. Also, a toolbox has been created, containing change management information and communication tools such as posters, postcards, reminders, patient information, infographics, etc. The toolbox is free available at toolbox-samen-beslissen-met-topics-sf.pdf (zorgvoorbeter.nl).

To empower older adults to prepare for a consultation and to share their priorities on personal health outcomes with health professionals, we adapted the layout of the patient preparatory tool to align with the implementation of the TOPICS-SF and in coordination with the Dutch patient association we aligned the layout to the national ‘Ask3questions’ campaign to enhance the recognizability for the Dutch older population. Furthermore, we developed a short, animated information film, to inform and motivate older adults to prepare for SDM with help of the TOPICS-SF. Also, similar as the online training platform for health professionals, a scenario with conversations with a virtual trainings ‘doctor’ was developed in co-creation with older adults (De Oefendokter). ‘De Oefendokter’ is free available in the same portal: https://samenbeslissen.dialoguetrainer.com/. Furthermore, the Dutch senior organization KBO-PCOB provide both online and offline information sessions to inform older adults and informal caregivers about SDM and the importance to prepare for a conversation with the health professional.

## Conclusions

This study shows that the SDM^MCC^ training for geriatricians improved discussion of goals, options, and decision making. In addition, it provides scope for improvement—discussing partnerships and the evaluation of the decision-making process could be reinforced. Furthermore, it might be valuable to use a preparatory tool to prepare and support the patient and caregiver; however, more attention should be given to integrating this tool in the consultation.

## Supplementary Information

**Additional file 1.** Patient preparatory tool.

**Additional file 2.** Description of the implementation of the SDM^MCC^ intervention.

**Additional file 3.** Adapted control preference scale.

**Additional file 4.** Baseline characteristics informal caregivers.

**Additional file 5.** Evaluation of use of the patient preparatory tool in the intervention group (n = 108).

**Additional file 6.** Observer OPTION^MCC^ scores with and without use of patient preparatory tool (intervention group).

**Additional file 7.** Observer OPTION^MCC^ scores for geriatricians, patients and informal caregivers (subgroup analysis of eight geriatricians).

## Data Availability

The datasets used and/or analyzed during the current study are available from the corresponding author on reasonable request. The preparatory tool can be downloaded for free in English and Dutch at https://www.vilans.org/app/uploads/2019/07/patient-brochure.pdf (English) and (Dutch). The SDM toolbox is free available at https://www.zorgvoorbeter.nl/zorgvoorbeter/media/documents/thema/persoonsgerichte-zorg/toolbox-samen-beslissen-met-topics-sf.pdf.

## Abbreviations

MCC: Multiple chronic conditions
SDM: Shared decision making
SDM^MCC^: Shared decision making for older adults with multiple chronic conditions
DICO: Decision making In Complex Older populations
AMC: Amsterdam Medical Center
MC SLV: Medical Center Slotervaart
PICS: Patients’ Perceived Involvement in Care Scale
DCS: Decisional Conflict Scale
