# Supplementary material for: Effects of a shared decision making intervention for older adults with multiple chronic conditions: the DICO study
Source: BMC Med Inform Decis Mak. 2023 Mar 1;23:42. doi: 10.1186/s12911-023-02099-2 (PMC9976432; doi:10.1186/s12911-023-02099-2)
Supplement: Supplementary file 1 — Additional file 1. Patient preparatory tool. [file 12911_2023_2099_MOESM1_ESM.docx]

**Additional file 1: Patient preparatory tool**


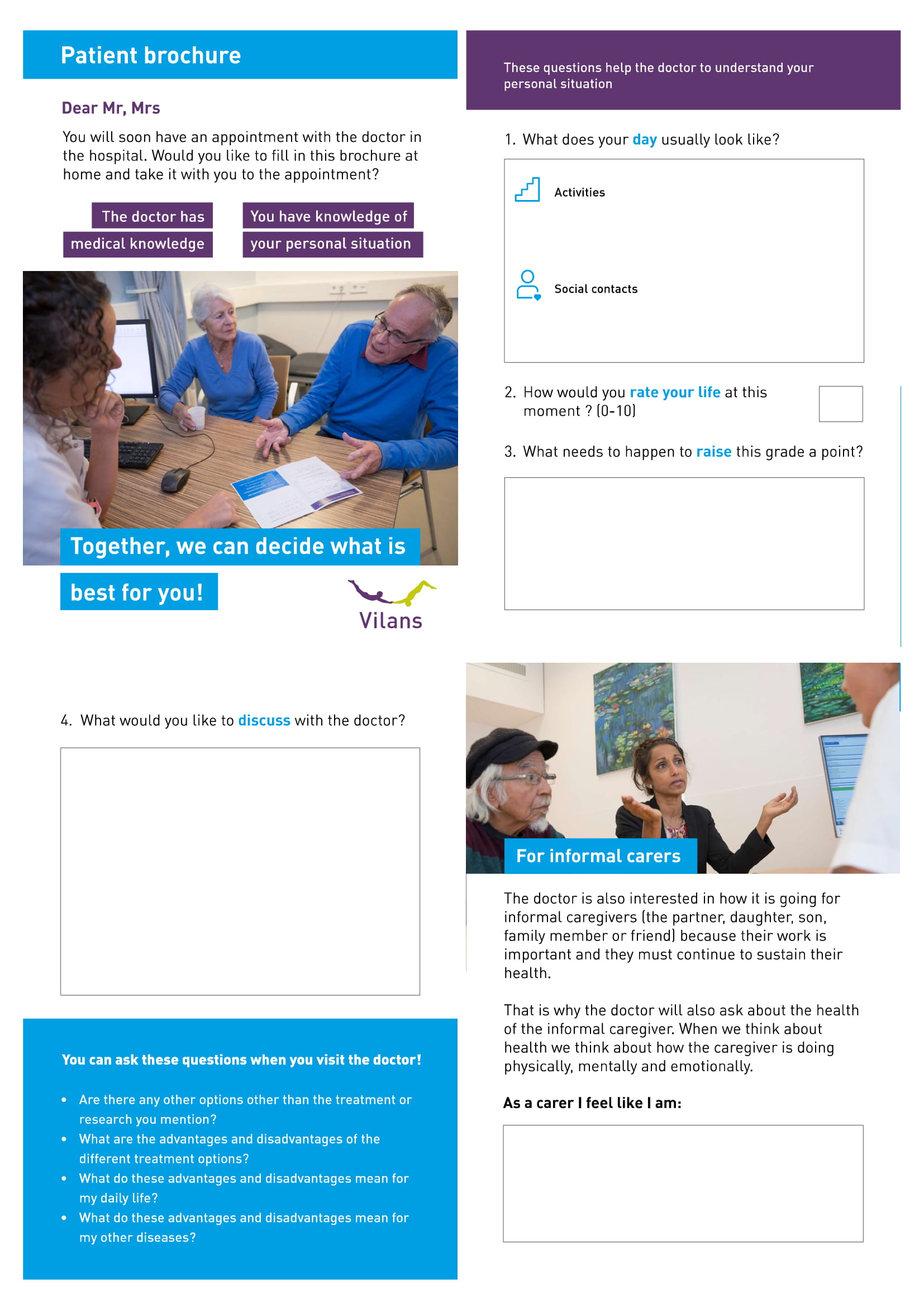


<https://www.vilans.org/app/uploads/2019/07/patient-brochure.pdf>
