# Supplementary material for: Effects of a shared decision making intervention for older adults with multiple chronic conditions: the DICO study
Source: BMC Med Inform Decis Mak. 2023 Mar 1;23:42. doi: 10.1186/s12911-023-02099-2 (PMC9976432; doi:10.1186/s12911-023-02099-2)
Supplement: Supplementary file 2 — Additional file 2. Description of the implementation of the SDMMCC intervention. [file 12911_2023_2099_MOESM2_ESM.docx]

**Additional file 2: Description of the implementation of the SDM^MCC^ intervention**

**1.Training for geriatricians**

In each hospital one 4-hour SDM^MCC^ training session was organized for which the geriatricians (n = 9) were invited. The SDM^MCC^ training was given by a teacher/researcher/general practitioner specialized in SDM with older adults with MCCs from the Radboud-university in Nijmegen. Theory about SDM in general and SDM with older adults was explained and demonstrated by several examples. In addition, a placemat with the steps of SDM with older adults with MCCs was distributed and the preparatory tool was shown and explained. A professional trainings-actor attended the meeting to provide training opportunities and the principle researcher guided the process. In the non-academic hospital, all five geriatricians followed the SDM^MCC^ training. In the academic hospital two geriatricians followed the 4-hour SDM^MCC^ training session, two geriatricians were not able to attend that day due to logistic reasons (1) and illness (1). Those two geriatricians received afterwards an adapted SDM^MCC^ training, that consisted of two informative video’s made by the trainer, about SDM in general and about the SDM^MCC^ model. This was followed by a meeting with the principal researcher so they could ask questions and discuss SDM. All geriatricians were offered a feedback session with the trainer to discuss the SDM process, using a videotape of one of their daily clinical consultations, six geriatricians were able to participate in this individual feed-back session.

**2. Preparatory tool for patients and informal caregivers**
The preparatory tool (patient folder) was sent by mail to all eligible patients and their informal caregivers (n=65) that were scheduled for a geriatric consultation in one of the two hospitals who wanted to participate in the study. In an information letter about the study, the purpose of the folder was explained and patients were requested to fill in the folder, if possible with their informal caregiver and bring it to the consultation.
