# Supplementary material for: Effects of a shared decision making intervention for older adults with multiple chronic conditions: the DICO study
Source: BMC Med Inform Decis Mak. 2023 Mar 1;23:42. doi: 10.1186/s12911-023-02099-2 (PMC9976432; doi:10.1186/s12911-023-02099-2)
Supplement: Supplementary file 3 — Additional file 3. Adapted control preference scale. [file 12911_2023_2099_MOESM3_ESM.docx]

**Additional file 3: Adapted Control Preference Scale**

Patients’ and informal caregivers’ preferred and perceived role in decision making was measured with the Control Preference Scale (CPS) [1, 2]*.* The CPS is a widely used tool, and assesses the level of control patients and clinicians want to assume in decision making. The original CPS consists of one item with a five-point scale [3]. The answer options range from the patient selecting its own treatment through a collaborative model to a scenario where the physician alone makes the decision [1]. In this study, the CPS was adapted to include the informal caregiver as a potential partner in decision making. The adapted scale contained seven response statements, which were divided among three categories: 1) an active role (patient- and/or informal caregiver-controlled), 2) a passive role (practitioner-controlled), and 3) a shared role (collaborative). The response statements from the adapted scale, and their category (active/passive/collaborative) are shown, for both the patients and the informal caregivers.

*Adapted CPS scale, pre-consultation questionnaire*.

| Response statement from adapted CPS | Preferred role of the patient | Preferred role of the informal caregiver |
| --- | --- | --- |
| I prefer that the patient himself/herself makes the final decision | Active | Passive |
| I prefer that the clinician makes the final decision | Passive | Passive |
| I prefer that the informal caregiver makes the final decision | Passive | Active |
| I prefer that the patient makes the final decision after seriously considering the clinician’s opinion | Active | Passive |
| I prefer that the patient makes the final decision after seriously considering the informal caregiver’s opinion | Active | Active |
| I prefer that the informal caregiver makes the final decision after seriously considering the clinician’s opinion | Passive | Active |
| I prefer that the patient, the informal caregiver, and the clinician share responsibility for the final decision | Collaborative | Collaborative |

*Adapted CPS scale, post-consultation questionnaire*.

| Response statement from adapted CPS | Preferred role of the patient | Preferred role of the informal caregiver |
| --- | --- | --- |
| The patient himself/herself has made the final decision during the consultation | Active | Passive |
| The clinician has made the final decision during the consultation | Passive | Passive |
| I prefer that the informal caregiver makes the final decision | Passive | Active |
| The patient has made the final decision during the consultation after seriously considering the clinician’s opinion | Active | Passive |
| The patient has made the final decision during the consultation after seriously considering the informal caregiver’s opinion | Active | Active |
|  |  |  |
| The informal caregiver has made the final decision during the consultation after seriously considering the clinician’s opinion | Passive | Active |
| The patient, the informal caregiver, and the clinician have shared responsibility for the final decision during the consultation | Collaborative | Collaborative |

**References**

1. Degner LF, Sloan JA, Venkatesh P: **The Control Preferences Scale**. *The Canadian journal of nursing research = Revue canadienne de recherche en sciences infirmieres* 1997, **29**(3):21-43.

2. Henrikson NB, Davison BJ, Berry DL: **Measuring decisional control preferences in men newly diagnosed with prostate cancer**. *Journal of psychosocial oncology* 2011, **29**(6):606-618.

3. Sung VW, Raker CA, Myers DL, Clark MA: **Treatment decision-making and information-seeking preferences in women with pelvic floor disorders**. *International urogynecology journal* 2010, **21**(9):1071-1078.
