# Supplementary material for: Effects of a shared decision making intervention for older adults with multiple chronic conditions: the DICO study
Source: BMC Med Inform Decis Mak. 2023 Mar 1;23:42. doi: 10.1186/s12911-023-02099-2 (PMC9976432; doi:10.1186/s12911-023-02099-2)
Supplement: Supplementary file 4 — Additional file 4. Baseline characteristics informal caregivers. [file 12911_2023_2099_MOESM4_ESM.docx]

**Additional file 4: Baseline Characteristics Informal Caregivers**

**Baseline Characteristics Informal Caregivers**

| **Characteristics** | **All Informal caregivers**  **(N=133)** | **Usual Care**  **Informal caregivers**  **(N^a^=68)** | **Intervention**  **Informal caregivers**  **(N^a^=65)** | **Intervention vs. usual care**  **informal caregivers**  **p=** |
| --- | --- | --- | --- | --- |
|  |  |  |  |  |
| Mean age in years (SD) | 65.6 (14.0) | 66.5 (13.1) | 64.2 (15.2) | .20 |
|  |  |  |  |  |
| Female sex (n, %) | 75 (57.7) | 43 (64.2%) | 32 (50.0) | .10 |
| Level of education |  |  |  | .12 |
| Low (n, %) | 5 (3.9) | 5 (7.5) | 0 (0) |  |
| Middle (n, %) | 78 (60.9) | 40 (59.7) | 38 (60.3) |  |
| High (n, %) | 45 (35.2) | 22 (32.8) | 23 (35.5) |  |
| Living situation |  |  |  | .59 |
| Independent, alone (n, %) | 14 (10.7) | 7 (10.4) | 7 (10.9) |  |
| Independent, with others (n,%) | 116 (88.5) | 60 (89.6) | 56 (87.5) |  |
| Home for the elderly (n, %) | 1 (.8) | 0 (0) | 1 (1.6) |  |
| Relation to the patient (n,%) |  |  |  |  |
| Husband/wife | 71 (56.3) | 38 (56.7) | 33 (52.4) |  |
| Daughter/Son | 41 (32.5) | 22 (32.8) | 19 (30.2) |  |
| Other relative | 5 (4.0) | 3 (4.5) | 2 (3.2) |  |
| Friend, neighbour, etc | 9 (7.1) | 4 (6.0) | 5 (7.9) |  |
|  |  |  |  |  |

*^a^ n varies slightly due to missing data*

*SD = standard deviation*

*< 0.05
**<0.01
***<0.001
