# Supplementary material for: Effects of a shared decision making intervention for older adults with multiple chronic conditions: the DICO study
Source: BMC Med Inform Decis Mak. 2023 Mar 1;23:42. doi: 10.1186/s12911-023-02099-2 (PMC9976432; doi:10.1186/s12911-023-02099-2)
Supplement: Supplementary file 5 — Additional file 5. Evaluation of use of the patient preparatory tool in the intervention group (n = 108). [file 12911_2023_2099_MOESM5_ESM.docx]

**Additional file 5: Evaluation of use of the patient preparatory tool in the intervention group (n=108)**

|  | Yes (n,%) |  |
| --- | --- | --- |
| *Patients:* Did you receive the preparatory tool? | 74 (68.5) |  |
| *Patients:* Did you complete the preparatory tool? | 56 (51.9) |  |
| *Patients:* Did you discuss the preparatory tool with your relatives? | 26 (24.1) |  |
| *Observers:* Was the preparatory tool used in the consultation? | 11 (10.2) |  |
|  | Patients that used the tool  (n=56) | Informal caregivers that used the tool (n=20) |
| What did you think of the preparatory tool?  good, clear or informative  confusing, difficult'  limited, too short'  not applicable to their situation  no opinion  other remarks (mainly logistic) | 64% 8% 7% 5% 5%  11% | 63% 7% 4% 19% 7% |
