# Supplementary material for: Effects of a shared decision making intervention for older adults with multiple chronic conditions: the DICO study
Source: BMC Med Inform Decis Mak. 2023 Mar 1;23:42. doi: 10.1186/s12911-023-02099-2 (PMC9976432; doi:10.1186/s12911-023-02099-2)
Supplement: Supplementary file 6 — Additional file 6. Observer OPTIONMCC scores with and without use of patient preparatory tool (intervention group). [file 12911_2023_2099_MOESM6_ESM.docx]

**Additional file 6: Observer OPTION^MCC^ scores with and without use of patient preparatory tool (intervention group)**

| 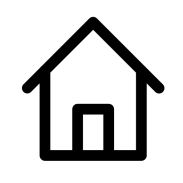 | Total OPTION score geriatricians (Transformed 0-100 score) (mean,sd) | Total OPTION score patients (mean,sd) | Total OPTION score informal caregivers (mean,sd) |
| --- | --- | --- | --- |
| Patient completed preparatory tool at home (n=56) | 39.3 (13.1) | 1.2 (.4) | .9 (.5) |
| Patient who **NOT** completed preparatory tool at home (n=52) | 39.4 (14.3) | 1.1 (.3) | 1.0 (.4) |
| 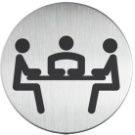 |  |  |  |
| Preparatory tool was used in consultation (n=11) | 38.6 (17.8) | 1.1 (.3) | .78 (.3) |
| Preparatory tool was **NOT** used in consultation (n=97) | 39.3 (13.7) | 1.1 (.4) | 1.0 (.5) |
