# Supplementary material for: Effects of a shared decision making intervention for older adults with multiple chronic conditions: the DICO study
Source: BMC Med Inform Decis Mak. 2023 Mar 1;23:42. doi: 10.1186/s12911-023-02099-2 (PMC9976432; doi:10.1186/s12911-023-02099-2)
Supplement: Supplementary file 7 — Additional file 7. Observer OPTIONMCC scores for geriatricians, patients and informal caregivers (subgroup analysis of eight geriatricians). [file 12911_2023_2099_MOESM7_ESM.docx]

**Additional file 7: Observer OPTION^MCC^ scores for geriatricians, patients and informal caregivers (subgroup analysis of eight geriatricians)**

|  | **Usual care**  **(n=81^a^)** | **Intervention**  **(86^a^)** | **P-value** | ***B* (95%CI)** |
| --- | --- | --- | --- | --- |
| **Geriatricians** |  |  |  |  |
| OPTION scores on subitems (mean. sd)  Range (0-4) |  |  |  |  |
| 1. Goal talk | 1.6 (1.0) | 2.0 (0.9) | 0.01^*^ | 0.39 (0.10;0.67) |
| 1. Option talk: present options | 1.7 (0.8) | 2.0 (0.6) | 0.01^*^ | 0.29 (0.08;0.50) |
| 1. Team talk: form partnership | 1.0 (1.1) | 0.6 (1.1) | 0.01^*^ | -0.43 (-0.75;-0.11) |
| 1. Option talk: discuss pro's and con's | 1.6 (0.9) | 1.9 (0.7) | 0.00^***^ | 0.39 (0.15;0.64) |
| 1. Decision talk: elicit preferences | 1.7 (1.2) | 2.1 (0.9) | 0.01^*^ | 0.42 (0.11;0.73) |
| 1. Decision talk: decide together | 1.5 (1.1) | 1.9 (0.9) | 0.01^*^ | 0.40 (0.09;0.70) |
| 1. Evaluation talk | 1.3 (1.0) | 0.9 (0.9) | 0.00^***^ | -0.43 (-0.70;-0.16) |
| Total OPTION score | 1.5 (0.8) | 1.6 (0.6) | 0.15 | 0.15 (-0.05;0.34) |
| **Patients** |  |  |  |  |
| OPTION scores on subitems (mean. sd)  Range (0-2) |  |  |  |  |
| 1. Goal talk | 1.1 (0.6) | 1.4 (0.6) | 0.00^***^ | 0.31 (0.13; 0.49) |
| 1. Option talk: present options | 1.0 (0.6) | 1.3 (0.6) | 0.00^***^ | 0.30 (0.12; 0.48) |
| 1. Team talk: form partnership | 0.7 (0.7) | 0.4 (0.7) | 0.00^***^ | -0.32 (-0.52; -0.12) |
| 1. Option talk: discuss pro's and con's | 1.0 (0.6) | 1.3 (0.6) | 0.00^***^ | 0.36 (0.18; 0.55) |
| 1. Decision talk: elicit preferences | 1.0 (0.7) | 1.5 (0.6) | 0.00^***^ | 0.50 (0.31; 0.69) |
| 1. Decision talk: decide together | 1.0 (0.7) | 1.4 (0.5) | 0.00^***^ | 0.39 (0.21; 0.57) |
| 1. Evaluation talk | 0.9 (0.7) | 0.7 (0.7) | 0.20 | -0.13 (-0.33; 0.07) |
| Total OPTION score | 0.9 (0.5) | 1.1 (0.4) | 0.00^***^ | 0.20 (0.07; 0.33) |
| **Informal caregivers** |  |  |  |  |
| OPTION scores on subitems (mean. sd)  Range (0-2) |  |  |  |  |
| 1. Goal talk | 1.2 (0.6) | 1.3 (0.6) | 0.24 | 0.14 (-0.10;0.38) |
| 1. Option talk: present options | 1.3 (0.7) | 1.2 (0.6) | 0.65 | -0.06 (-0.30;0.19) |
| 1. Team talk: form partnership | 1.0 (0.8) | 0.2 (0.5) | 0.00^***^ | -0.77 (-1.03;-0.51) |
| 1. Option talk: discuss pro's and con's | 1.2 (0.7) | 1.3 (0.7) | 0.43 | 0.11 (-0.15;0.37) |
| 1. Decision talk: elicit preferences | 1.2 (0.7) | 1.4 (0.6) | 0.02^*^ | 0.31 (0.06;0.56) |
| 1. Decision talk: decide together | 1.1 (0.7) | 1.4 (0.6) | 0.02^*^ | 0.28 (0.04;0.51) |
| 1. Evaluation talk | 0.9 (0.7) | 0.6 (0.7) | 0.03^*^ | -0.28 (-0.54;-0.03) |
| Total OPTION score | 1.1 (0.5) | 1.1 (0.4) | 0.72 | -0.03 (-0.20;0.14) |

^a^ n varies slightly due to missing data
*< 0.05
**<0.01
***<0.001
